# Influence of Dead Cells Killed by Industrial Biocides (BAC and DBNPA) on Biofilm Formation

**DOI:** 10.3390/antibiotics13020140

**Published:** 2024-01-31

**Authors:** Ana C. Barros, Diogo A. C. Narciso, Luis F. Melo, Ana Pereira

**Affiliations:** 1 LEPABE—Laboratory for Process Engineering, Environment, Biotechnology and Energy, Faculty of Engineering, University of Porto, Rua Dr. Roberto Frias, 4200-465 Porto, Portugal; acbarros@fe.up.pt (A.C.B.); diogo.narciso@tecnico.ulisboa.pt (D.A.C.N.); lmelo@fe.up.pt (L.F.M.); 2 ALiCE—Associate Laboratory in Chemical Engineering, Faculty of Engineering, University of Porto, Rua Dr. Roberto Frias, 4200-465 Porto, Portugal; 3 CERENA—Centro Recursos Naturais e Ambiente, Department of Chemical Engineering, Instituto Superior Técnico, Universidade de Lisboa, Avenida Rovisco Pais 1, 1049-001 Lisbon, Portugal

**Keywords:** biofilm prevention, dead cells, killed cells, benzalkonium chloride (BAC), 2,2-dibromo-3-nitrilopropionamide (DBNPA), mechanism of antimicrobial action, biofilm structure

## Abstract

Industrial biocides aim to keep water systems microbiologically controlled and to minimize biofouling. However, the resulting dead cells are usually not removed from the water streams and can influence the growth of the remaining live cells in planktonic and sessile states. This study aims to understand the effect of dead *Pseudomonas fluorescens* cells killed by industrial biocides—benzalkonium chloride (BAC) and 2,2-dibromo-3-nitrilopropionamide (DBNPA)—on biofilm formation. Additionally, the effect of different dead/live cell ratios (50.00% and 99.99%) was studied. The inoculum was recirculated in a Parallel Plate Flow Cell (PPFC). The overall results indicate that dead cells greatly affect biofilm properties. Inoculum with DBNPA–dead cells led to more active (higher ATP content and metabolic activity) and thicker biofilm layers in comparison to BAC–dead cells, which seems to be linked to the mechanism of action by which the cells were killed. Furthermore, higher dead cell ratios (99.99%) in the inoculum led to more active (higher culturability, metabolic activity and ATP content) and cohesive/compact and uniformly distributed biofilms in comparison with the 50.00% dead cell ratio. The design of future disinfection strategies must consider the contribution of dead cells to the biofilm build-up, as they might negatively affect water system operations.

## 1. Introduction

Biocidal programmes in most industrial water treatment processes aim to keep water systems microbiologically controlled rather than to eradicate all the microorganisms. An effective microbial control should consider the antimicrobial mechanism of action of the biocides, not only because a badly designed antimicrobial programme can lead to antimicrobial resistance [1,2,3] but it might affect bacteria recovery after biocidal exposure. For example, Barros et al. [4,5] showed that two industrially used biocides with different mechanisms of action (benzalkonium chloride (BAC) and 2,2-dibromo-3-nitrilopropionamide (DBNPA)) can damage bacteria to different extents and that previously dead-labelled bacteria recover their functional processes to significant levels when optimum planktonic growth conditions are replenished (resembling, for example, a failure of biocidal dosing in a real water system).

According to Flemming [6], even when 99.99% of microorganisms are killed in the pre-treatment stage, the remaining ones will still be able to colonise a surface, proliferate, and contribute to the formation or settling of biofilms. The characteristics of such biofilm layers may adversely affect the operation of water systems. It is also known that dead cells, apart from serving as a nutrient reservoir for live cells, can also display other features, such as communicate with their neighbouring cells by releasing specific molecules that will act as a “danger signal” [7,8,9,10,11,12] and, depending on the molecule, will induce a different response of the neighbouring cells, and may induce changes in gene expression [12]. It is widely recognised that cells injured by distinct mechanisms of antimicrobial action may differ in their behaviour and lead to different results in terms of biofilm formation [13,14,15]. 

So, understanding how killed/injured cells affect biofilm development is important to further mitigate the undesirable microbial (re)growth and operational impact of biofilms. To date, only a few studies have specifically addressed the impact of dead cells on biofouling and most have focused on the use autoclave–dead cells [16,17,18,19,20]. The main parameters analysed were the culturable and total cells, ATP and EPS concentration, permeate flux, and solute rejection.

Therefore, this study aims to bring forward new perspectives on the use of biocides in water treatment processes, with an emphasis on the role of dead cells and the mechanism by which they were killed, on biofilm properties, by evaluating how inoculum containing cells killed by BAC and DBNPA in different dead/live cell ratios affect the microbial features of a young (5 days) *Pseudomonas fluorescens* biofilm. The work combines information from: (i) bacterial culturability, viability and metabolic activity; (ii) mesoscale imaging (through OCT—Optical Coherence Tomography); and (iii) exopolysaccharide quantification, to better link the bacterial features to the biofilm structure.

## 2. Results

### 2.1. The Impact of Dead Cells on Biofilm Formation

Five-day-old *Pseudomonas fluorescens* biofilms, formed from an inoculum of dead cells mixed with live cells, were sampled and analysed in terms of the culturability, membrane integrity, cellular energy, metabolic activity, biofilm thickness and extracellular polymeric substances (EPS) concentration. Nutrients were provided so that live cells would not depend on dead cells as nutrients source. Dead cells were killed by biocides with different mechanisms of action (BAC and DBNPA) and their effects tested at different dead/live ratios (50.00% and 99.99%). Three independent experiments have been accomplished for each tested condition (two biocides at two dead/live ratios). 

**Figure 1:**
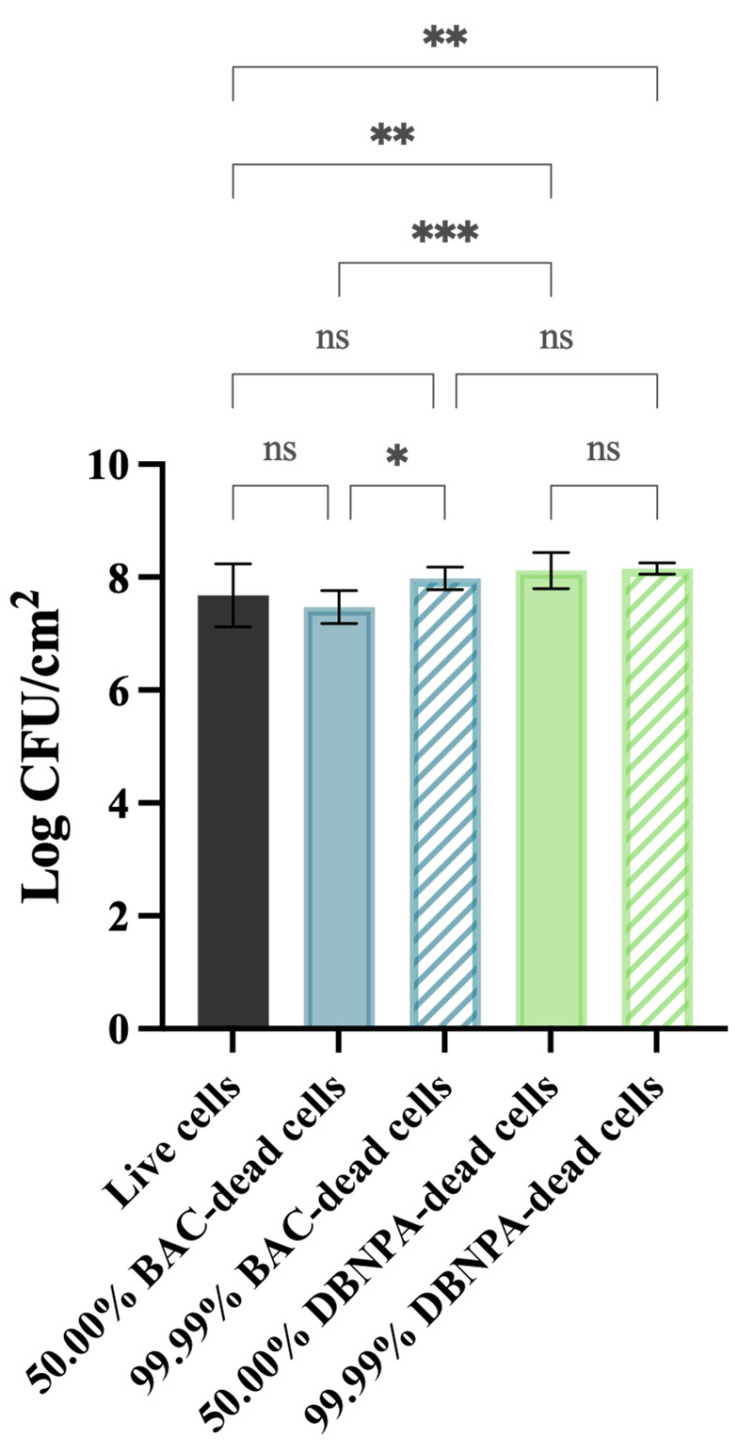
Culturability (in log CFU/cm^2^) of the biofilm cells formed on PVC coupons of the PPFC under different dead/live cell ratios. ‘ns’ indicates not significant (*p* > 0.05), whereas the asterisks indicate statistical significance (* *p* < 0.05; ** *p* < 0.01; and *** *p* < 0.001) using Dunn’s multiple comparisons test. The means ± SD of three independent experiments with four replicates (coupons) are presented.

Figure 1 shows that the cells’ culturability was higher for biofilms from DBNPA-killed cells than for the control (live cells), regardless (*p* < 0.01) of the dead cell ratio (50.00% or 99.99%). On the other hand, biofilms’ culturability from BAC-exposed cells showed a more diversified behaviour: (i) the biofilm cells culturability from 50.00% BAC-dead cells was slightly lower (*p* > 0.05) than the control (live cells); and (ii) biofilms formed by 99.99% dead/live cells presented a higher number of culturable cells when compared to the control (live cells) (*p* > 0.05) and to the 50.00% BAC–dead cells case (*p* < 0.05).

**Figure 2:**
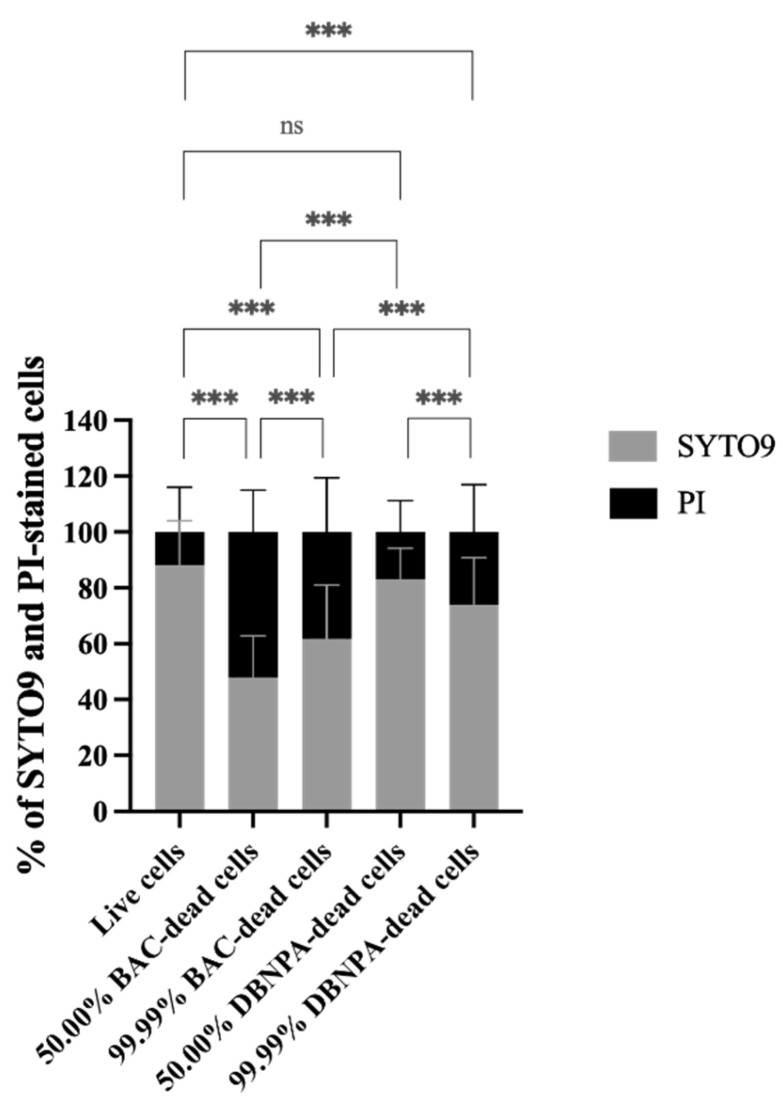
Membrane integrity in terms of the percentage of SYTO9 and propidium iodide (PI) uptake by *Pseudomonas fluorescens* biofilm cells formed on PVC coupons of the PPFC under different dead/live cell ratios. ‘ns’ indicates not significant (*p* > 0.05), whereas the asterisks indicate statistical significance (*** *p* < 0.001) using Dunn’s multiple comparisons test. The means ± SD of three independent experiments with four replicates (coupons) are presented.

The membrane integrity was evaluated in terms of the SYTO9 and PI uptake percentages (see Figure 2). Most samples presented statistically significant (*p* < 0.001) higher PI uptake percentages than the control, except the 50.00% DBNPA–dead cells’ biofilms (*p* > 0.05).

Interestingly, the biofilm build-up from BAC–dead cells showed higher PI uptake when compared to DBNPA, regardless of the dead cell ratio.

Comparison of the SYTO9 and PI uptake values showed that, except for one case (50.00% BAC–dead cells), higher SYTO9 uptake percentages were observed, suggesting that the biofilms were mainly composed by cells with intact membranes.

**Figure 3:**
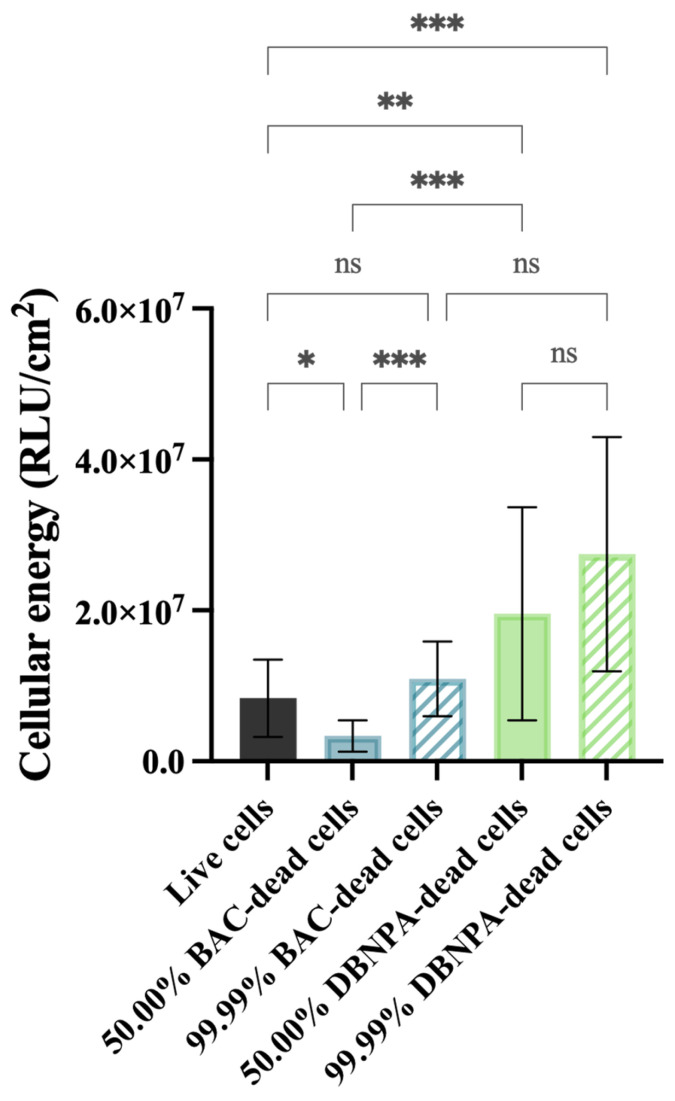
Cellular energy (in RLU/cm^2^) of the biofilm cells formed on PVC coupons of the PPFC under different dead/live cell ratios. RLU—relative light units. ‘ns’ indicates not significant (*p* > 0.05), whereas the asterisks indicate statistical significance (* *p* < 0.05; ** *p* < 0.01; and *** *p* < 0.001) using Dunn’s multiple comparisons test. The means ± SD of three independent experiments with four replicates (coupons) are presented.

The biofilms showed different cellular energy—Figure 3—depending on the characteristics of the dead cells in the inoculum (BAC vs. DBNPA; 50.00% vs. 99.99% dead/live ratio). While the biofilms from 50.00% BAC–dead cells presented lower ATP levels (*p* < 0.05) when compared to live cells, the biofilms formed by DBNPA–dead cells (at 50.00 and 99.99%) showed significantly increased (*p* < 0.01) ATP levels compared to the control. 

Comparison of the luminescence values between the biofilms formed in the presence of dead cells killed by the same biocide showed an increase from 50.00% to 99.99%; however, such differences were only statistically significant for BAC—dead cells (*p* < 0.001).

**Figure 4:**
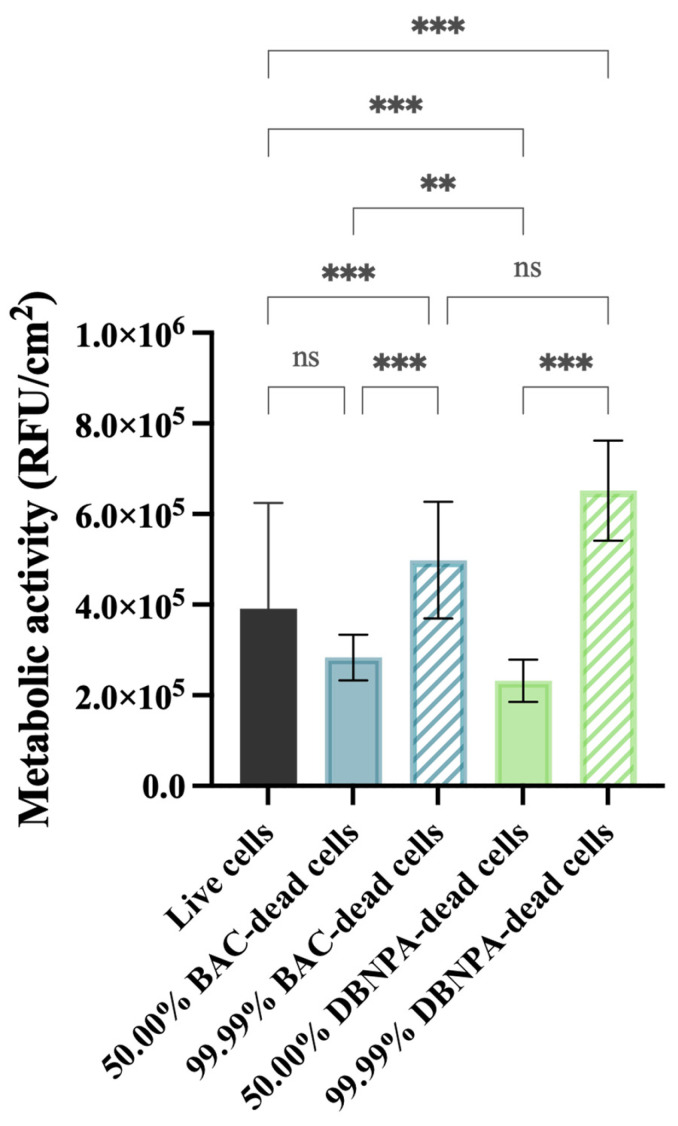
Metabolic activity (in RFU/cm^2^) of the biofilm cells formed on PVC coupons of the PPFC under different dead/live cell ratios. RFU—relative fluorescence units. ‘ns’ indicates not significant (*p* > 0.05), whereas the asterisks indicate statistical significance (** *p* < 0.01 and *** *p* < 0.001) using Dunn’s multiple comparisons test. The means ± SD of three independent experiments with four replicates (coupons) are presented.

The metabolic activity of the cells was measured in terms of the fluorescence intensity (RFU/cm^2^) and these data are presented in Figure 4.

All the tested conditions showed statistically significant differences (*p* ≤ 0.01) comparing to the control (3.9 × 10^5^ RFU/cm^2^), except for the biofilms formed from 50.00% BAC–dead cells (*p* > 0.05).

It is interesting to note that the biofilms formed from cultures with 50.00% dead cells showed lower fluorescence intensity when compared to the control (3.98 × 10^5^ RFU/cm^2^), regardless of the biocide used. Furthermore, when the reactor was inoculated with a higher number of dead cells (99.99%), higher fluorescence was observed in comparison to the corresponding 50.00% dead biofilms (*p* < 0.001).

### 2.2. Biofilm Thickness and Structure

**Figure 5:**
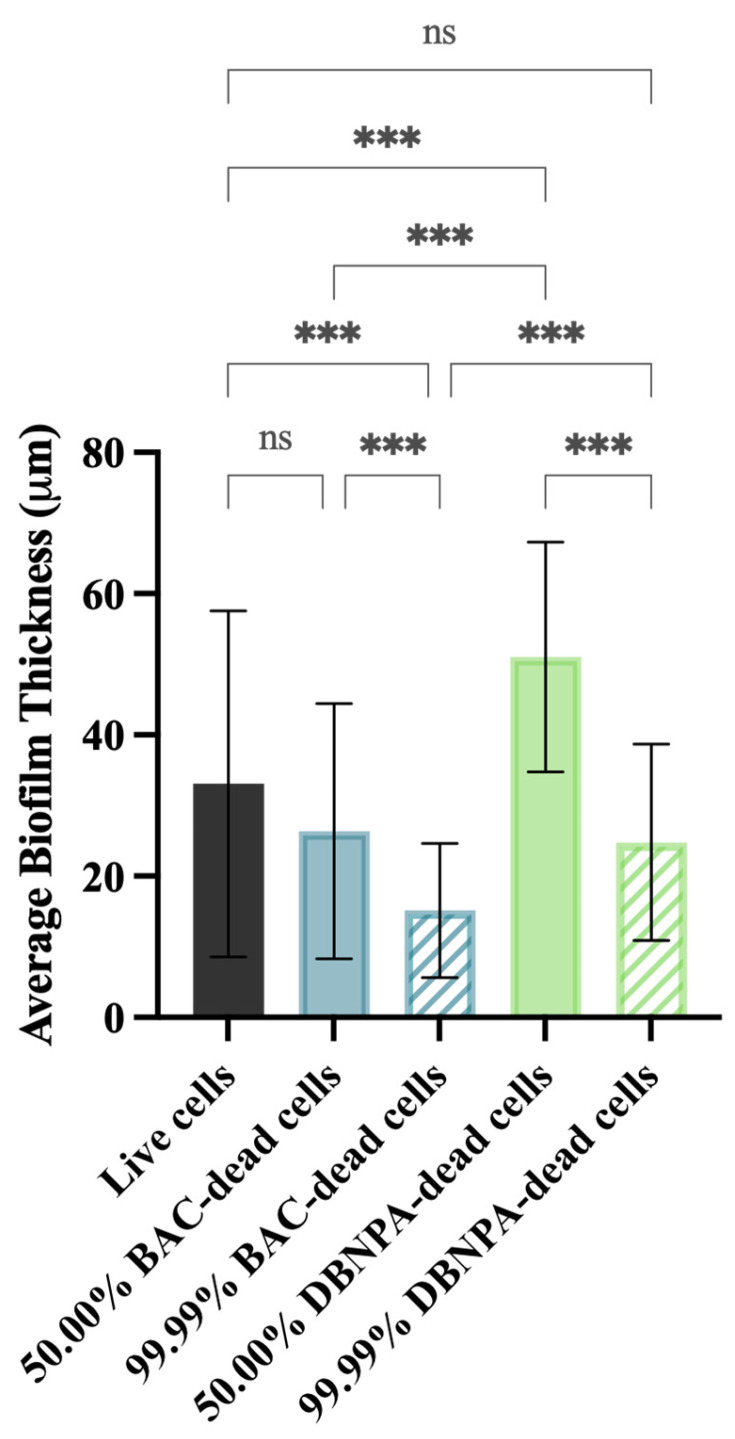
Average biofilm thickness formed on PVC coupons of the PPFC under different dead/live cell ratios. ‘ns’ indicates not significant (*p* > 0.05), whereas the asterisks indicate statistical significance (*** *p* < 0.001) using Dunn’s multiple comparisons test. The means ± SD of three independent experiments with four replicates (coupons) are presented.

The biofilm thickness and structure were evaluated using optical coherence tomography (OCT). In general, the thickness of the biofilms from the dead cell inoculum was lower than the biofilms from live cells (33 µm)—Figure 5. The exception was biofilms from 50.00% DBNPA–dead cells, which presented the highest average biofilm thickness (51 µm).

In addition, there was a significant (*p* < 0.001) biofilm thickness reduction from 50.00% to 99.99% dead cells, regardless of the biocide used to kill the cells. For instance, thickness values of 26 µm (50.00% BAC–dead cells), 15 µm (99.99% BAC–dead cells), 51 µm (50.00% DBNPA–dead cells), and 25 µm (99.99% DBNPA–dead cells) were found. Moreover, higher thickness values were observed for the biofilms from DBNPA–dead cells (at 50.00 and 99.99%) when comparing to the BAC ones.

**Figure 6:**
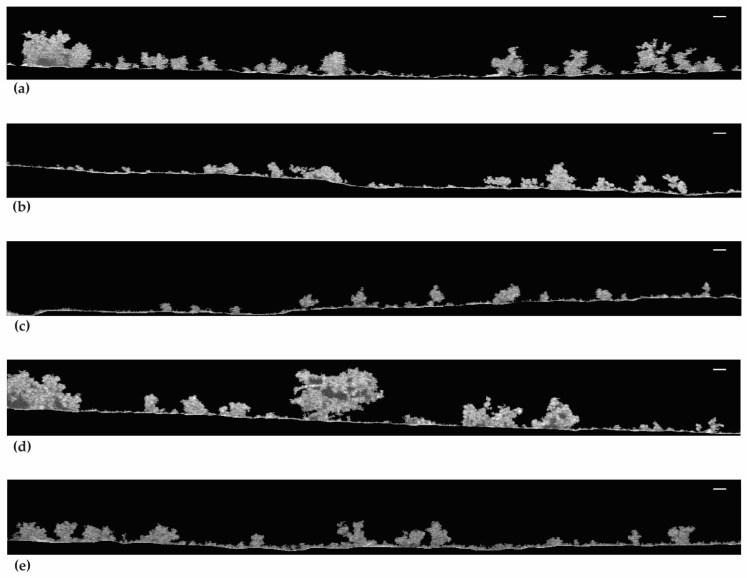
OCT observations of the biofilm formed on PVC coupons of the PPFC under different dead/live cell ratios: (**a**) live cells; (**b**) 50.00% BAC–dead cells; (**c**) 99.99% BAC–dead cells; (**d**) 50.00% DBNPA–dead cells; and (**e**) 99.99% DBNPA–dead cells. Images obtained using BISCAP software and show biofilm vertical stacks. Scale bar = 100 µm.

The 2D-OCT images in Figure 6 show the biofilm structure at the mesoscale. Figure 6a,d,e show that the biofilms from live cells and 50.00% and 99.99% DBNPA–dead cells have a more heterogeneous matrix. Regarding Figure 6b, the biofilms from 50.00% BAC–dead cells have a smaller three-dimensional structure (the protuberances) and seem more compact than the previous ones (Figure 6a,d,e). Additionally, thinner biofilms with flat/smooth surfaces were observed when the inoculum contained 99.99% BAC–dead cells (Figure 6c).

In brief, increasing dead cell ratio in the inoculum resulted in thinner and more homogeneous biofilms. Overall, inoculum with dead cells from different biocides led to different biofilm structures.

The OCT images allowed the calculation of an additional variable: the compaction parameter. As defined by Narciso et al. [21], the compaction parameter represents the ‘ratio between the total number of continuous biomass pixels in the biofilm structure and the total number of pixels between the bottom and top interfaces’. Such a variable indicates (in a normalised form, values range from 0 to 1) how compact is the biofilm structure: a compaction parameter of 1 would indicate a biofilm that has no empty spaces, while values close to zero represent an imaginary ‘empty’ (no biomass) biofilm [21]. Figure S1 (Supplementary Material) shows that increasing the ratio of dead/live cells increased the compaction parameter, regardless of the killing method, particularly when using the inoculum with 99.99% dead cells (*p* < 0.001 when compared to the control).

### 2.3. EPS Quantification

**Figure 7:**
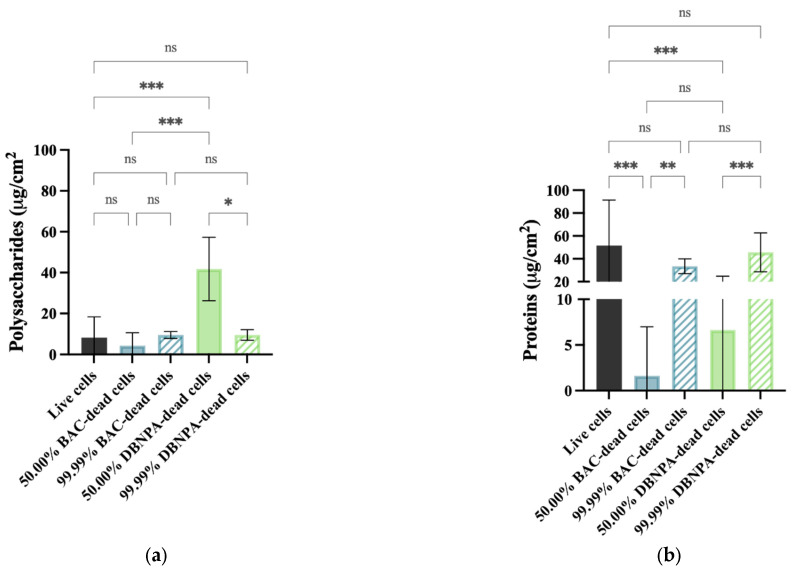
Polysaccharide (**a**) and protein content (**b**) of isolated EPS from biofilms formed on PVC coupons of the PPFC under different dead/live cell ratios. ‘ns’ indicates not significant (*p* > 0.05), whereas the asterisks indicate statistical significance (* *p* < 0.05; ** *p* < 0.01; and *** *p* < 0.001) using Dunn’s multiple comparisons test. The means ± SD of three independent experiments with four replicates (coupons) are presented.

The EPS composition was analysed according to its content of proteins and polysaccharides (Figure 7). Regarding polysaccharides (Figure 7a), statistically significant changes (*p* < 0.05) were found for the 50.00% cultures from DBNPA–dead cells in comparison to the live cells. Also, the differences between the 50.00% and the correspondent 99.99% dead biofilms were only statistically significant for DBNPA (*p* < 0.05).

The EPS biofilms from live cells had a higher protein content (Figure 7b) than all the other samples, although these differences were much less pronounced for all the cases with 99.99% dead cells (*p* > 0.05). There was an increase (*p* ≤ 0.01) in the protein concentration when comparing the 50.00% and their correspondent 99.99% dead cells (for both biocides).

## 3. Discussion

In a former study [5], the authors concluded that the recovery pattern of planktonic dead cells depended on the biocides (BAC vs. DBNPA) they were previously exposed to. The current work investigates if the presence of differently killed cells in the inoculum also affects the properties of the young (5 days) biofilms they build up by tackling the following points: (a) to understand how the biocides behind cells death affect (or not) the biofilm properties; (b) whether the biofilm properties differences are comparable, per biocide, to the planktonic dead cells recovery (the former study [5]); and finally, (c) to evaluate how different dead/live cells ratios (*v*/*v* ratio) impact biofilm properties.

### 3.1. How Does the Planktonic Cells’ Killing Method Affect the Biofilm Properties?

The overall results suggest that the biofilm properties are different depending on the presence of BAC- or DBNPA-killed cells in the inoculum and on the dead/live cell ratio. For the 50.00% dead cells ratio, there is a statistically significant difference (*p* < 0.001) between the biofilms formed from BAC- and from DBNPA-killed cells for all the evaluated biofilm properties, while for 99.99%, such significant differences (*p* < 0.001) were only found for the PI uptake and thickness. Globally, it also seems that biofilms formed from DBNPA-killed cells tend to be more microbiologically active and to have higher thicknesses than the ones formed from BAC-dead cells. In fact, when comparing these biofilm results with the ones obtained for dead cell recovery in the planktonic state (discussed in Barros et al. [5]), they follow similar patterns regarding whether the cells were previously exposed to BAC or DBNPA. Such differences, in the planktonic study, were found to be related to the underlying mechanism of action of each biocide: BAC interferes with the cytoplasmatic membrane and might promote intracellular content release [22,23], while DBNPA interferes with key metabolic pathways of the cell but does not significantly affect the membrane stability [24,25]. 

In the planktonic cell recovery studies, this justified why BAC-killed cells upon recovery showed higher PI uptake percentages than DBNPA despite the TEM micrographs showed apparently intact cell membranes in the latter case (Barros et al. [5]). Also, the much higher ATP content of DBNPA cells’ recovery comparing to BAC ones seems to be associated with the inhibition of high energy-consuming processes by the DBPNA mechanism of action, which favours the accumulation of ATP in cells [26,27]. 

As mentioned, the trends observed for the microbiological properties in planktonic studies are also present in the current study for the biofilms. This suggests that biofilms are also affected by the underlying biocidal mechanism action by which dead cells in the inoculum were previously killed. But, why would biofilm features depend on the dead cells’ killing method and why would these features be comparable to the dead cells’ recovery in the planktonic state? Arguably, dead cells, when present in the inoculum, are integrated into the biofilms and become a relevant component of it. For example, the PI uptake for BAC-killed cells biofilms is higher than the one observed for biofilms formed from DBNPA-killed ones, suggesting that there is a significant amount of cells with injured membranes in the biofilm (allowing the PI to penetrate and stain the cells’ membrane). Another example is the higher ATP levels observed for DBNPA-dead cells comparing to BAC ones, which is possibly linked to the ability that cells exposed to DBNPA have to enter the VBNC state and accumulate ATP [26,27]. These results suggest that damaged cells are a significant part of the biofilm even after 5 days. 

Another aspect that stands out in the present work is that dead cells contribute to different extents to the biofilm development—significantly higher thicknesses were found for biofilms from DBNPA-killed cells rather than BAC ones. Indeed, the increased thickness of DBNPA biofilms is arguably another consequence of the accumulation of ATP inside the DBNPA-exposed cells [26,27]. Increased ATP reserves allow cells inside the biofilm to recover more quickly, contributing to the higher thickness of biofilms from DBPNA–dead cells. On the other hand, biofilms formed from BAC-exposed cells need more time to recover from membrane injuries and so, comparably, they show lower thicknesses.

It is, though, intriguing that the way cells were previously killed (BAC or DBNPA) is more noticeable in the experiments where the inoculum contained the highest concentrations of live cells. For instance, 50.00% corresponds to 10^7^ CFU/mL of live cells and 99.99% just to 10^4^ CFU/mL, as described in Section 4.3. Indeed, for the 50.00% dead/live ratios, there are statistically significant differences for all the analysed parameters. It seems that the presence of dead cells in the 50.00% cases negatively affected the viability of the cells (CFUs, ATP and metabolic activity) and the total EPS (polysaccharides + proteins) production. Accordingly, Schink et al. [28] found that the presence of a higher number of *Escherichia coli* UV-killed cells promoted the growth of live cells rather than just focussing on their own survival. Interestingly, they also observed an increase in the lag phase time with increasing the amount of live cells (and decreasing the number of dead cells) [28]. Since the lag phase of bacterial growth refers to the initial period when bacteria are adjusting to their new environment and preparing for active reproduction [29], it is not surprising to observe an increase in the lag phase time in bacteria upon exposure to antimicrobial agents. Furthermore, the different patterns observed for the 50.00% dead cell biofilms are particularly prominent in the case of the DBNPA-killed cell biofilms, even in comparison to the control (10^8^ CFU/mL of live cells). Zayed et al. [15] demonstrated that the exposure of bacterial cells to heat resulted in increased biofilm formation (total cells) in comparison to hydrogen peroxide-killed cells and to the control of live biofilms.

### 3.2. The Effects of Inoculum with Different Dead/Live Cell Ratios on Biofilm Build-Up

Our results indicate that inoculum containing dead cells promote the build-up of biofilms with higher cell culturability, cellular energy and metabolic activity than biofilms formed with live cells only. These effects become more pronounced as the dead cell ratio increases, which agrees with former studies [18,20]. Kim et al. (2015), while studying biofouling caused by live and dead cells (killed by autoclave), observed that the ATP concentration from membranes fouled with dead cells was 1.93 times higher than when fouled with live cells. Necrotrophic growth may explain the high number of bacteria observed after heat treatment of a water system [13,30,31]. Accordingly, Yang et al. [20] concluded that the presence of autoclave–dead cells greatly contributed to the increase in cell density and activity in the feed water of a membrane system. 

Regarding the biofilm thicknesses, it decreased from the 50.00% to 99.99% dead cell ratio. In the 99.99% case, the cells might have spent more energy and resources to recover from their injured state instead of using them for biofilm maturation, which probably justifies the high energy levels reflected by the cellular energy and metabolic activity values.

Also, the protein content in the EPS was positively correlated to the dead cell percentage: biofilm EPS from 99.99% of dead cells had higher protein content than 50.00% (*p* < 0.01), although in a lower amount than the biofilm from live cells (*p* > 0.05). Yang et al. [20] also found that the fouling of membranes with dead cells had a significant increase in the protein concentration. On the other hand, there are no significant differences between the polysaccharides content when comparing to the control (live cells), except for biofilms from 50.00% DBNPA–dead cells. When considering the total EPS (the sum of polysaccharides and proteins), there is a higher EPS content for 99.99% dead cell ratio. Given the important nature of the EPS in regulating the adhesive/cohesive interactions between cells and the mediation of initial cell adhesion [18,32], it might be speculated that biofilms formed by higher amounts of dead cells (99.99%) seem to be more cohesive/compact since they present a higher total EPS content, lower thicknesses, more uniformly distributed biomass and higher compaction parameters. This pattern was observed for BAC and for DBNPA.

Finally, it is worth noting that even though, in the present study, nutrients were continuously fed into the system, a detailed study about the effect of the inoculum size (relative volume of live cells) was not considered. Also, a control containing dead cells only could be performed to compare to the live cells control. According to some authors [12,14,33], the effect of dead cells on biofilm build-up is not affected by the cellular density of the live cells. However, others [29,34] point out that the way experiments are initiated might affect the biofilm formation and microbiological features. So, future research should focus on the effect that the following aspects might have on the final biofilm characteristics: (a) different cellular densities (e.g., 10^4^ and 10^7^ CFU/mL) of live cells only; and (b) dead cells only (in different volumes). Future work should also consider the imaging of the live/dead cells in the biofilm, for example, through Confocal Laser Scanning Microscopy, and the comparison of the effect of the differently killed cells on the build-up of biofilms with different ages (e.g., young vs. mature biofilms).

## 4. Materials and Methods

### 4.1. Biocides

Two biocides were used in this study: benzalkonium chloride (BAC, 50% (*w*/*w*), Thor Specialties Inc., Shelton, CT, USA) and 2,2-dibromo-3-nitrilopropionamide (DBNPA, 10% (*w*/*w*) of active ingredient, Enkrott^®^, S.A, Sintra, Portugal). The biocide solutions were freshly prepared in sterile ultrapure water.

### 4.2. Microorganisms and Culturing Conditions

*Pseudomonas fluorescens* isolated from a drinking water distribution system and identified via 16S rRNA gene sequencing [35] was selected for this study. *P. fluorescens* was cultured overnight in batch cultures using a nutrient medium composed of 5 g/L glucose (Merck, Darmstadt, Germany), 2.5 g/L peptone (Merck, Darmstadt, Germany), and 1.25 g/L yeast extract (Merck, Darmstadt, Germany) in a 0.02 M phosphate buffer with pH 7 (KH_2_PO_4_; Na_2_HPO_4_—Chem-Lab NV, Zedelgem, Belgium) at 30 °C under agitation at 120 rpm (Agitorb 200 ICP, Norconcessus, Ermesinde, Portugal).

### 4.3. Obtaining Dead Bacterial Cells 

An overnight growth culture of *P. fluorescens* was centrifuged (Eppendorf centrifuge 5810 R, Eppendorf, Hamburg, Germany) at 3100× *g* (4000 rpm), washed, and resuspended in saline solution—0.85% (*v*/*v*) to an optical density of approximately 0.2 ± 0.02 (~1.2 × 10^8^ CFU/mL). To obtain dead cells, *P. fluorescens* cells (final volume of 500 mL) were exposed to bactericidal concentrations of the selected biocides for 30 min in a shaking incubator at 160 rpm and 25 °C. BAC–dead cells resulted from the contact of bacteria with 160 mg/L of BAC, while DBNPA–dead cells were previously exposed to 35 mg/L of DBNPA. These concentrations were chosen since in a previous work, Barros et al. [4] found that concentrations above the minimum bactericidal concentrations were needed to obtain dead cells. After biocide exposure, the cells were centrifuged and washed twice in saline solution to remove the remaining biocide. 

**Figure 8:**
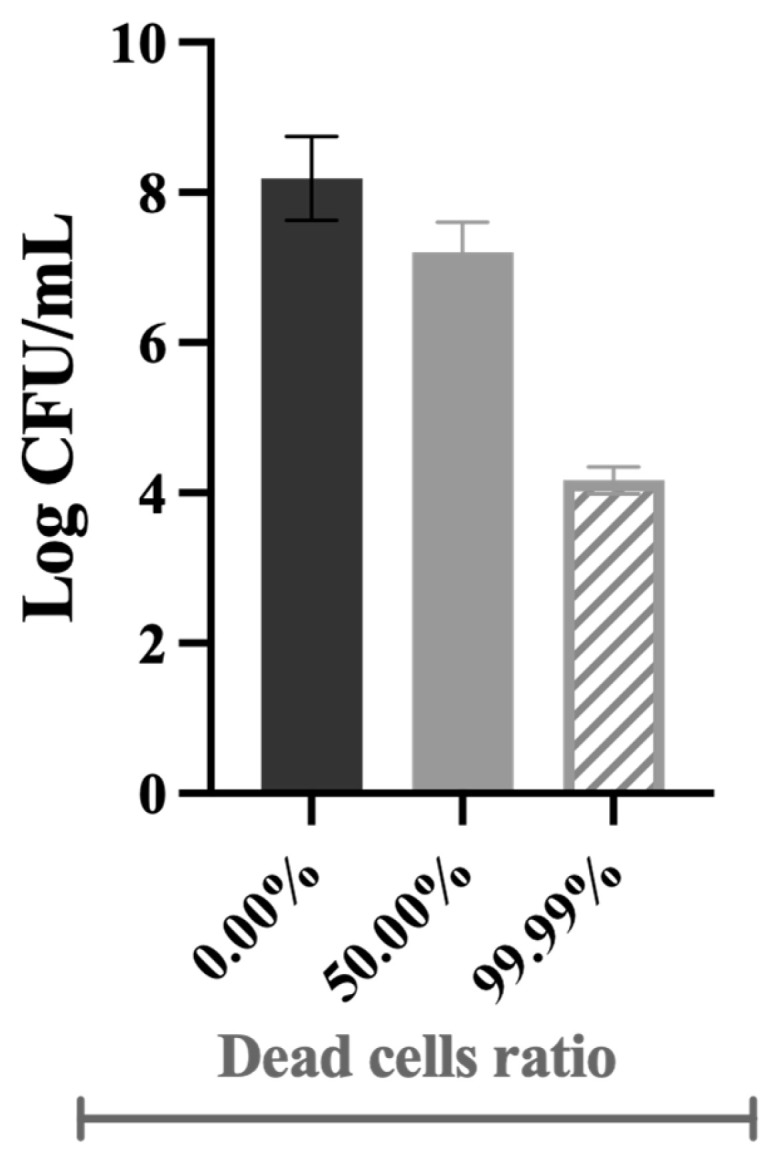
Illustrative representation of the culturability of bacterial cells in the inoculum at different dead cell ratios.

Samples with various ratios of dead cells (volume/volume ratio) were obtained by mixing live cells with dead cells (killed by biocides)—Figure 8. Since not all the dead/live cell ratios could be tested, 99.99% was chosen to show that the efficacy of most disinfection strategies is overestimated, i.e., even if 99.99% of bacteria are killed in pre-treatment systems, a few colonies will survive and be able to grow and colonise other surfaces [6]. The 50.00% ratio was selected to have a dead cell concentration in the middle range. 

The control corresponds to live cells only (0.00% dead cells). For instance, the 50.00% dead cell ratio is composed of 250 mL suspensions of live cells with 250 mL of the obtained dead cells. All the concentrations tested were validated prior to the chemostat inoculation by analysing the culturability, membrane integrity, cellular energy, and metabolic activity of planktonic cells. For example, the 50.00% and 99.99% dead cells correspond to ~3.4 × 10^7^ and 1.6 × 10^4^ CFU/mL, respectively, as illustrated in Figure 8.

### 4.4. Biofilm Formation on a PPFC System

**Figure 9:**
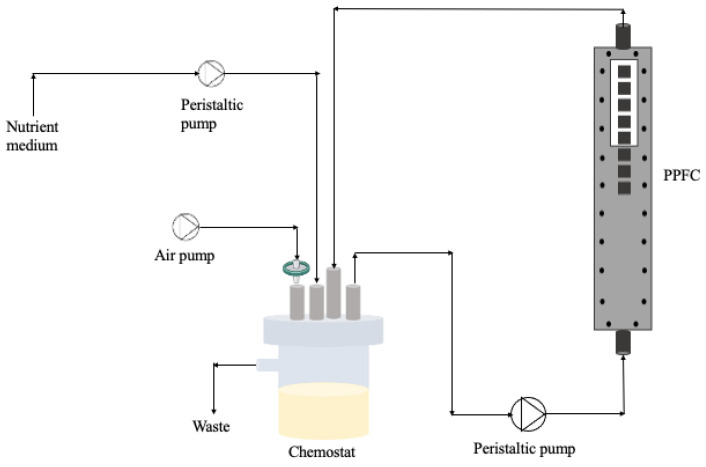
Schematic representation of the flow cell system.

For the biofilm development, a parallel plate flow cell (PPFC; Neves & Neves, Metalomecânica Lda., Trofa, Portugal) similar to the one described by Moreira et al. [36] was used. The PPFC is a small polyvinyl chloride (PVC) flow cell with a cross-section of 0.8 × 1.6 cm and a length of 25.42 cm. Also, the PPFC has a transparent window on the top plate, which allows biofilm visualisation during the experiment (Figure 9). Additionally, the PPFC has recesses in the bottom plate allowing the surface incorporation of 8 coupons that will be in contact with bacteria at specific hydrodynamic conditions. In this study, PVC coupons (0.9 × 0.9 × 0.2 cm) were chosen as the surface material for biofilm formation.

Bacterial suspensions with different ratios of dead cells (from Section 4.3) were placed in a 385 mL chemostat (Neves & Neves, Metalomecânica Lda., Trofa, Portugal) and recirculated through the PPFC system at 0.16 m/s (see Figure 9). So, the system was inoculated with dead/live cells at the beginning of the experiments and there was no continuous input of bacteria/biocide over time. The residence time of the bacteria was 10 h and the Reynolds number in the PPFC was 417, meaning that the flow was laminar. The chemostat was aerated and magnetically agitated.

To ensure that biofilm prevailed over planktonic cells, after 2 h, a 100× diluted nutrient medium was fed continuously at a dilution rate of 0.1/h. The period for biofilm formation was 5 days. Three independent experiments were performed for each condition.

### 4.5. Biofilm Analysis

#### 4.5.1. Biofilm Visualisation Using Optical Coherence Tomography

*In-situ* imaging of the biofilm was performed using Optical Coherence Tomography (OCT). The equipment consisted of an OCT (Thorlabs Ganymede GmbH, Dachau, Germany) with a central wavelength of 930 nm, equipped with a 5× telecentric scan lens (Thorlabs LSM03BB). A refractive index of 1.4 was selected as biofilms are mainly composed of water (refractive index: 1.3) [37]. A minimum of 12 images were analysed to ensure the reliability of the results. The BISCAP version 1.0 (Biofilm Imaging and Structure Classification Automatic Processor) software was used to analyse the acquired images. This freeware software is available at https://github.com/diogonarciso/BISCAP (downloaded on 26 January 2022) and further details on the image processing are described in Narciso et al. [21]. In brief, firstly all the pixels at the substratum (surface) are identified, exploring the fact that these are usually very bright. Then, a threshold intensity is calculated and all the pixels binarised accordingly (biomass vs. background). At last, using two complementary pixel continuity checks, the full biofilm structure and all the pixels at the boundary with the liquid bulk are identified. All the steps are fully automatic. BISCAP delivers multiple image outputs for detailed biofilm visualisation and analysis. Among all the biofilm structural parameters that can be obtained using this software, in the present study, only the average thickness (μm) and the compaction parameter results will be presented. The mean biofilm thickness can be calculated as the length between the interfaces (top and bottom) that outline the biofilm architecture [21] from the 2D OCT images.

#### 4.5.2. Sampling

The coupons were placed in tubes with 3 mL of saline solution (85% (*v*:*v*) NaCl). The biofilm was removed by submitting the coupons to an ultrasonic treatment (45 kHz) for 2 min (VWR ultrasonic cleaner USC1700T, VWR, Leuven, Belgium), followed by 2 min of vortexing at 3000 rpm (ZX3 vortex mixer, VELP Scientifica^®^, Usmate (MB), Italy). The obtained biofilm suspension was used for the extracellular polymeric substance (EPS) isolation, culturability, membrane integrity, cellular energy, and metabolic activity determination. Four coupons were used for the EPS determination, and the other four coupons were used for the viability tests.

#### 4.5.3. Culturability

To determine the culturability of the cells, the drop plate method [38] was used. Samples were serially diluted (in 8.5 g/L NaCl) to an appropriate cell density and plated on plate count agar (PCA). After 24 h of incubation at 30 °C, cell enumeration was carried out. The results are shown as the logarithm of the colony forming units per cm^2^ (log CFU/cm^2^).

#### 4.5.4. Cell Membrane Integrity

The membrane integrity of the cells was evaluated as fully described by Barros et al. [4]. Briefly, the cell suspension was properly diluted, stained for 7 min with the nucleic acids SYTO9^TM^ and propidium iodide (PI), available from the LIVE/DEAD^®^ Baclight^TM^ kit (Invitrogen/Molecular Probes, Eugene, OR, USA), and filtered through a 0.2 μm Nucleopore^®^ (Whatman, Middlesex, UK) black polycarbonate membrane. Afterwards, the membranes were prepared for microscope observation (LEICA DMLB2; Leica Microsystems Ltd., Heerbrugg, Switzerland). The biofilm cells were observed under a 100× immersion oil objective of a CCD camera coupled with a mercury lamp HBO/100W/3 (λ_excitation_ = 480–500 nm and λ_emission_ = 485 nm; Chroma 61000-V2 DAPI/FITC/TRITC). The cell numbers were estimated from the counts of a minimum of 15 fields of view (6.03 × 10^−5^ cm^2^) and the results were presented as the SYTO 9 and PI uptake percentages.

#### 4.5.5. Cellular Energy

Adenosine triphosphate (ATP) measurements were performed using a BacTiter-Glo^TM^ Microbial Cell Viability Assay (Promega, Madison, WI, USA) according to the manufacturer’s instructions. Briefly, 100 µL of sample was mixed with an equal volume of the BacTiter-Glo^TM^ reagent in a 96-well white microtiter plate. Afterwards, the luminescence (in relative luminescence units—RLU) was recorded using a FLUOstar^®^ Omega microtiter plate reader (BMG Labtech, Ortenberg, Germany). Data were calculated as RLU/cm^2^.

#### 4.5.6. Metabolic Activity

The metabolic activity of the cells was evaluated using a resazurin reduction assay as described in Barros et al. [4]. In short, microbial suspensions were mixed with resazurin in 96-well black microtiter plates (protected from light). After 24 h, the fluorescence (in relative fluorescence units-RFU) was measured using a FLUOstar^®^ Omega microtiter plate reader (λ_excitation_ = 570 nm and λ_emission_ = 590 nm; BMG Labtech, Ortenberg, Germany). Data are presented as RFU/cm^2^.

#### 4.5.7. EPS Extraction

For the EPS extraction, the biofilms were resuspended in 3 mL of extraction buffer (2 mM Na_3_PO_4_, 2 mM NaH_2_PO_4_, 9 mM NaCl and 1 mM KCl, pH 7), mixed a with a cation exchange resin (Dowex^®^, Sigma-Aldrich, Steinheim, Germany) and shaken for 4 h at 400 rpm (RT 15 power multi-position magnetic stirrer, IKAMAG, Staufen, Germany) and 4 °C [39]. Bacteria were separated from the EPS through centrifugation at 3100× *g* (4000 rpm) for 5 min [40].

##### Protein Quantification

The proteins were determined via the Pierce method using bovine serum albumin (BSA) as the standard. In short, a working solution of the BCA reagents (Pierce™ BCA Protein Assay Kit product no. 23225, Thermo Fisher Scientific, Carlsbad, CA, USA) was mixed according to the manufacturer’s instructions. Next, 50 μL of each sample (or standard) were pipetted into a 96-well plate and 200 μL were added to each well. Then, the plate was shaken for 30 s and incubated in the dark at 37 °C for 30 min. Afterwards, the absorbance at 562 nm was recorded using a microtiter plate reader (SPECTROstar Nano, BMG LABTECH, Ortenberg, Germany). Each sample was measured in triplicate, and the data are presented as μg/cm^2^.

##### Polysaccharide Quantification

The polysaccharides were determined via the phenol-sulfuric acid method [41] using glucose as the standard. In brief, 250 μL of each sample (or standard) was mixed with 500 μL of a 5% phenol solution and 2.5 mL of concentrated sulfuric acid. After the reaction, the solution was cooled to room temperature and the absorbance at 490 nm recorded using a spectrophotometer (V-1200, VWR, Leuven, Belgium). Each sample was measured in duplicate, and the data are presented as μg/cm^2^.

### 4.6. Statistical Analysis

Statistical analysis was performed using Graphpad Prism version 9.0.1 for macOS software (GraphPad Software, San Diego, CA, USA). Data were analysed using the non-parametric Kruskal–Wallis test, followed by Dunn’s multiple comparison test. Statistical calculations were based on a confidence level ≥ 95%. All the data were expressed as the means ± SD of three independent experiments with four replicates each. Asterisks indicate significant differences between groups, * *p* < 0.05, ** *p* < 0.01, *** *p* < 0.001, ns—not significant.

## 5. Conclusions

Most industrial water treatment processes rely on the use of biocides to ensure microbiological control and to prevent biofouling. Nevertheless, dead/injured cells, cell debris and leaked intracellular compounds are usually not removed in such water systems and might end up within the biofilm, causing major operational problems. 

The present study deepens the understanding of the presence of dead cells in the inoculum used for biofilm formation by analysing: (a) inoculum containing cells killed by two industrial biocides, BAC and DBNPA, which have different antimicrobial mechanisms; and (b) inoculum with different dead/live ratios of cells.

Overall, our results suggest that: (i) the mechanism of biocidal action behind cells’ inactivation affects the final biofilm characteristics, particularly when more live cells are present (50.00% dead/live cell ratio); (ii) the presence of DBNPA–dead cells in the inoculum leads to more active (higher ATP content and metabolic activity) and thicker biofilm layers in comparison to BAC–dead cells; and (iii) the presence of higher dead cell ratios in the inoculum (99.99%) leads to more active biofilms (higher culturability, metabolic activity and ATP content) and lower thicknesses, regardless of the killing method; these biofilms tend to be more cohesive/compact and more uniformly distributed across the adhesion surface when compared to the 50.00% dead cell ratio and to the control.

From an operational point-of-view in water treatment systems, it is clear that the contribution of dead cells to the biofilm build-up must be taken into consideration when designing the biocidal strategy and the appropriate pre-treatment stages.

## Data Availability

Data are contained within article and Supplementary Materials.

## Supplementary Materials

The following supporting information can be downloaded at https://www.mdpi.com/article/10.3390/antibiotics13020140/s1, Figure S1: Compaction parameters of biofilms formed on PVC coupons of the PPFC under different dead cell ratios.
