# Supplementary material for: Influence of Dead Cells Killed by Industrial Biocides (BAC and DBNPA) on Biofilm Formation
Source: Antibiotics (Basel). 2024 Jan 31;13(2):140. doi: 10.3390/antibiotics13020140 (PMC10885908; doi:10.3390/antibiotics13020140)
Supplement: Supplementary file 1 [file antibiotics-13-00140-s001.zip › antibiotics-2820463-supplementary.pdf]

antibiotics

MDPI

Supplementary Materials

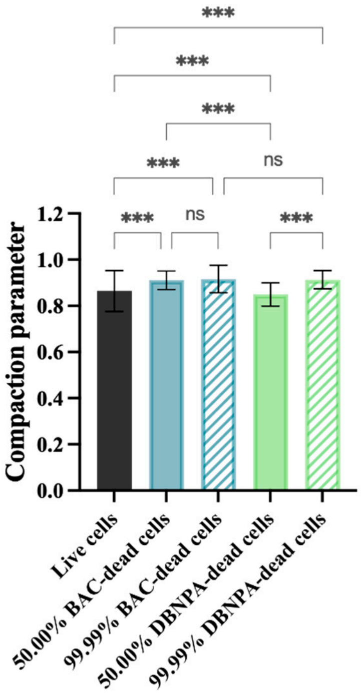

Figure S1. Compaction parameter of biofilms formed on PVC coupons of the PPFC under different dead cells ratios. 'ns' indicates not significant ($p > 0.05$), whereas the asterisks indicate statistical significance (*** $p < 0.001$) using Dunn's multiple comparisons test. The means ± SD of three independent experiments with four replicates (coupons) are presented.
